# Morphological, Molecular and Phylogenetic Characterization of *Ceratomyxa nemiptera* sp. nov. (Myxozoa: Ceratomyxidae) Infecting *Nemipterus virgatus* Houttuyn, 1782 in the East China Sea

**DOI:** 10.3390/ani16020166

**Published:** 2026-01-07

**Authors:** Pingping Li, Yang Zhou, Xiaoping Tan, Yuanjun Zhao, Chengzhong Yang

**Affiliations:** Animal Biology Key Laboratory of Chongqing Education Commission of China, Chongqing Key Laboratory of Conservation and Utilization of Freshwater Fishes, College of Life Sciences, Chongqing Normal University, Chongqing 401331, China; 2024110513038@stu.cqnu.edu.cn (P.L.); tigerzy@163.com (Y.Z.); tanxp406@126.com (X.T.); zhaoyuanjuncqnu@126.com (Y.Z.)

**Keywords:** Myxosporea, taxonomy, gallbladder infection, SSU rDNA, species diversity

## Abstract

A newly discovered myxosporean parasite was described from the gallbladder of *Nemipterus virgatus* Houttuyn, 1782 collected from the East China Sea. Mature myxospores are crescent-shaped with shell valves that taper gradually toward rounded ends. Each myxospore contained two sub-spherical polar capsules located near the anterior end, closely aligned along the suture line. The mature myxospores measured 6.2 ± 0.6 (5.4–6.9) μm in length and 44.8 ± 4.6 (38.5–53.1) μm in thickness. Polar capsules measured 2.8 ± 0.2 (2.4–3.1) μm in length and 2.3 ± 0.2 (1.9–2.6) μm in width, with polar filaments coiled in 2–3 turns. The small subunit ribosomal DNA (SSU rDNA) sequence of *Ceratomyxa nemiptera* sp. nov. was distinct from all known myxosporeans, showing the highest similarity (93.56%) and the shortest genetic distance (0.0637) with *Ceratomyxa arcuata* Thélohan, 1892. The phylogenetic analysis revealed that *C. nemiptera* sp. nov. was positioned within a later-diverging lineage, forming a sister-group relationship with a clade containing *C. arcuata* and *Ceratomyxa cretensis* Kalatzis, Kokkari & Katharios, 2013. This is the first report of a *Ceratomyxa* species infecting *N. virgatus*.

## Simple Summary

Myxosporean parasites are a large group of tiny organisms that infect both marine and freshwater fish, and identifying their species can be tricky due to their small size and variable shapes. *Ceratomyxa*, a large genus of myxosporeans, mostly infects the gallbladders of marine fish, and scientists now use a combination of morphological characteristics, infection site, host and genetic data to identify species of *Ceratomyxa*. *Nemipterus virgatus*, an economically important fish in southeastern Chinese coastal fisheries, is a known myxosporean host, but little is known about these parasites in the East China Sea. In our study, we found a new *Ceratomyxa* species (*Ceratomyxa nemiptera* sp. nov.) in the gallbladders of *N. virgatus* from the East China Sea. This new parasite has a crescent-shaped body with two spherical polar capsules near its front end, and its genetic material is distinct from other known myxosporeans, being most closely related to *Ceratomyxa arcuata*. This is the first time a *Ceratomyxa* species has been found infecting *N. virgatus*, adding to current knowledge of myxosporean diversity in the East China Sea and helping protect this important commercial fish.

## 1. Introduction

Myxosporeans (Myxosporea Bütschli, 1881) are a highly diverse and globally distributed group of microscopic metazoan parasites infecting both marine and freshwater fishes [1,2]. To date, more than 3000 species have been formally described, and new taxa continue to be discovered as molecular tools advance [3,4,5]. These parasites are characterized by complex life cycles, typically involving both vertebrate and invertebrate hosts, and by their relatively simple morphology [1,3]. The small size and limited number of diagnostic features, together with morphological plasticity observed in some species, often obscure interspecific boundaries and make species identification and classification particularly challenging [6,7,8].

Within Myxosporea, the genus *Ceratomyxa* Thélohan, 1892 represents one of the largest and most morphologically diverse lineages, currently comprising over 300 valid species [9,10,11]. Mature myxospores of *Ceratomyxa* are elongate, usually crescentic or arcuate, and sometimes ovoid or even sub-spherical. Conical or sub-hemispherical shell valves exceed one-half of the axial diameter of the myxospore in length and are usually pliable. Polar capsules are sub-spherical, positioned close to the suture line at the anterior pole of the myxospore, but exceptionally open laterally from the central suture line. Sporoplasm is generally binucleate; however, the presence of two uninucleated sporoplasms has also been observed in some species [12,13]. Species of *Ceratomyxa* predominantly infect the gallbladders of marine teleosts, although a few have been recorded in freshwater environments [9,14].

Traditionally, species identification within the genus *Ceratomyxa* has been based primarily on morphological characteristics and morphometry. However, some members of this genus often exhibit considerable morphological plasticity under different hosts or environmental conditions, making morphology alone insufficient for reliable taxonomic identification [6,15,16]. Consequently, modern taxonomic approaches emphasize an integrative framework that combines morphological characteristics, parasitic traits, and molecular data [7,10,11,14].

*Nemipterus virgatus* Houttuyn, 1782, belonging to the family Nemipteridae (order Perciformes), is a warm-water, medium-sized demersal fish distributed mainly in tropical and subtropical regions of the western Pacific and Indian Oceans. It is an economically important species along the southeastern coast of China, playing a significant role in regional marine fisheries [17]. Previous studies have reported that *N. virgatus* serves as a host to two myxosporean parasites, *Kudoa megacapsula* Yokoyama & Itoh, 2005 (family Kudoidae) and *Unicapsula trigona* Li, Tamemasa, Zhang & Sato, 2019 (family Trilosporidae), which may influence the physiological condition and commercial value of the host [18,19].

The East China Sea is one of the most fish-rich marine regions in the world, characterized by exceptionally high ichthyofaunal diversity. To date, a total of 442 fish species from this area have been recorded, belonging to more than 300 genera, 152 families, and 29 orders [20,21]. Despite this remarkable diversity, studies on myxosporean parasites of fishes from the East China Sea remain relatively limited. According to currently available reports, only a small number of myxosporean species have been documented from this region, comprising approximately 12 species infecting about 10 marine fish hosts. These parasites predominantly infect the gallbladder, with only occasional records from other organs such as the alimentary tract [7,10,22,23,24,25,26,27,28]. In recent years, we have conducted a series of surveys on myxosporeans from the East China Sea, during which a new *Ceratomyxa* species was identified and described using an integrative approach.

## 2. Materials and Methods

### 2.1. Sample Collection and Morphological Analysis

**Figure 1:**
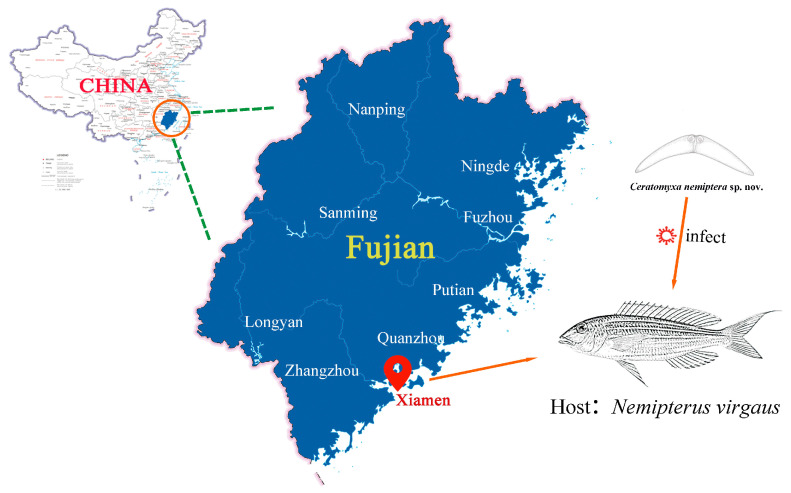
Map showing sampling locality for host of *Ceratomyxa nemiptera* sp. nov. The figure created by authors and the base map of China was sourced from the Standard Map Service System of the Ministry of Natural Resources of the People’s Republic of China (http://bzdt.ch.mnr.gov.cn/, accessed on 3 January 2026) with the approval number of GS(2019)1671.

Eighteen specimens of Golden threadfin bream, *Nemipterus virgatus* Houttuyn, 1782 were obtained from nearshore fishing vessels operating off the coast of Xiamen, China, in July 2018. All specimens were captured from the East China Sea. Sampling locations are shown in Figure 1. Upon arrival at the laboratory, each specimen was carefully examined for myxosporean infections. A thorough inspection was conducted on various organs and tissues, including the skin, fins, gills, musculature, hepatopancreas, intestine, spleen, heart, gallbladder, and urinary bladder, as well as on body fluids such as bile, blood, and urine [11]. Isolated myxospores were collected for both morphological and molecular analyses. Species identification and specimen processing followed the procedures described by Zhao et al., 2001 [29]. Fresh myxospores were examined and measured under a Leica DM6000B light microscope at ×1000 magnification (Leica, Wetzlar, Germany). All measurements were taken from 30 mature myxospores for the new *Ceratomyxa* species and are expressed in micrometres (μm) as the mean ± standard deviation, followed by the range in parentheses. Line drawings of the new species were prepared using CorelDRAW 11.0 software.

The morphological comparison between the new species and other ceratomyxids was conducted based on spore morphology, with reference to all valid species of the genus *Ceratomyxa*. Particular attention was given to species infecting hosts of identical or related taxonomy and occurring in the same or geographically similar regions.

### 2.2. DNA Extraction and Amplification

Myxospores obtained from host bile and preserved in 95% ethanol were isolated by centrifugation. Genomic DNA was extracted from parasite-rich gallbladder bile using the DNeasy Tissue Kit (QIAGEN, Hilden, Germany) following the manufacturer’s instructions. The small subunit ribosomal DNA (SSU rDNA) fragment was amplified using primers ERIB1 (5′-ACCTGGTTGATCCTGCCAG-3′) and ERIB10 (5′-CTTCCGCAGGTTCACCGCAGG-3′) [30], followed by a nested PCR using the primer pairs CERAss1-F (5′-CGCTCCAAGTGAGTGCCATC-3′)/CERAss1-R (5′-ACCTGTTATTGCCACGCTTCC-3′) and CERAss2-F (5′-GAAGCGTGGCAATAACAGGTC-3′)/CERAss2-R (5′-AGAGGCAGAGACGTATTCAACA-3′) [11]. Each PCR reaction was carried out in a final volume of 25 μL, containing 0.5 μL of each primer, 1.5 μL of DNA template, 12.5 μL of PCR Master mix, and ddH_2_O to the final volume. The PCR cycling conditions consisted of an initial denaturation at 94 °C for 2 min, followed by 35 cycles of 94 °C for 30 s, 56 °C for 30 s, and 72 °C for 1 min, with a final extension at 72 °C for 10 min. PCR products were visualized by agarose gel electrophoresis and purified amplicons were ligated into the pMD19-T vector (TaKaRa, Otsu, Japan). The recombinant plasmids were then transformed into *Escherichia coli* strain DH5α. Eight positive transformants were cultured, plasmids were purified, and four clones were selected for sequencing using an ABI Prism 377 DNA Sequencer (Applied Biosystems, Carlsbad, CA, USA). The resulting SSU rDNA sequences of myxospores were assembled using ContigExpress software (Vector NTI Suite 6.0, Invitrogen, Carlsbad, CA, USA), and the consensus sequence was deposited in the National Center for Biotechnology Information (NCBI) GenBank database for public access.

### 2.3. Molecular and Phylogenetic Analysis

Sequence similarity and genetic distance were calculated between the newly obtained SSU rDNA sequence and those showing the highest identity in the NCBI BLAST 2.8.1 search, as well as with the morphologically similar species. Pairwise sequence similarities were computed using the MAFFT program from the EMBOSS suite [31], and genetic distances (p-distance) were calculated in MEGA 11 [32].

A phylogenetic tree was constructed based on a total of 130 SSU rDNA sequences, including the newly obtained sequence and closely related sequences retrieved from GenBank. Two species from the genus *Enteromyxum*, *E. fugu* Tun, Yokoyama, Ogawa & Wakayabashi, 2000 (GenBank accession No. AY520573) and *E. scophthalmi* Palenzuela, Redondo & Alvarez-Pellitero, 2002 (AF411335), were selected as outgroup taxa to root the tree. All sequences were aligned using MEGA 11 [32], and highly variable, poorly aligned, or rapidly evolving regions were removed using Gblocks 0.91b [33]. Phylogenetic relationships were inferred using both Bayesian Inference (BI) and Maximum Likelihood (ML) methods. The BI was performed in MrBayes 3.1.2 [34] under a general time-reversible (GTR) model with gamma-distributed rate variation. Four Markov chains were run simultaneously for three million generations, sampling every 200 generations, and the first 25% of samples as burn-in. The ML analysis was conducted on the CIPRES portal (https://www.phylo.org/, accessed on 3 January 2026) using RAxML [35] with the GTR+gamma model, and bootstrap support values were calculated from 1000 replicates. The resulting BI and ML phylogenetic trees were visualized and edited using FigTree v1.4.2 and Adobe Photoshop.

## 3. Results

### 3.1. Taxonomic Summary

Phylum: Cnidaria Hatschek, 1888

Subphylum: Myxozoa Grassé, 1970

Class: Myxosporea Bütschli, 1881

Order: Bivalvulida Shulman, 1959

Family: Ceratomyxidae Doflein, 1899

Genus: *Ceratomyxa* Thélohan, 1892

*Ceratomyxa nemiptera* sp. nov.

ZooBank registration number: urn:lsid:zoobank.org:pub:82CB82BB-AB69-4D63-B4E1-AA44755EC980.

Type host: *Nemipterus virgatus* Houttuyn, 1782 (Perciformes: Nemipteridae).

Type locality: Coastal waters near Xiamen (118°12′34″ E, 24°21′20″ N), East China Sea (Figure 1).

Infection site: Gallbladder.

Date of sampling: July 2018.

Prevalence: Of 18 *N. virgatus* specimens examined, two were infected (11.1%).

Deposition of materials: Specimens of *N. virgatus* (mounted in glycerin-alcohol-formalin; accession number TZ2018070006) were deposited in the collection centre of Animal Biology Key Laboratory of Chongqing Education Commission of China, Chongqing Normal University, Chongqing, PR China.

Etymology: The species epithet nemiptera refers to the genus of the type host, *Nemipterus*.

### 3.2. Morphological Description

**Figure 2:**
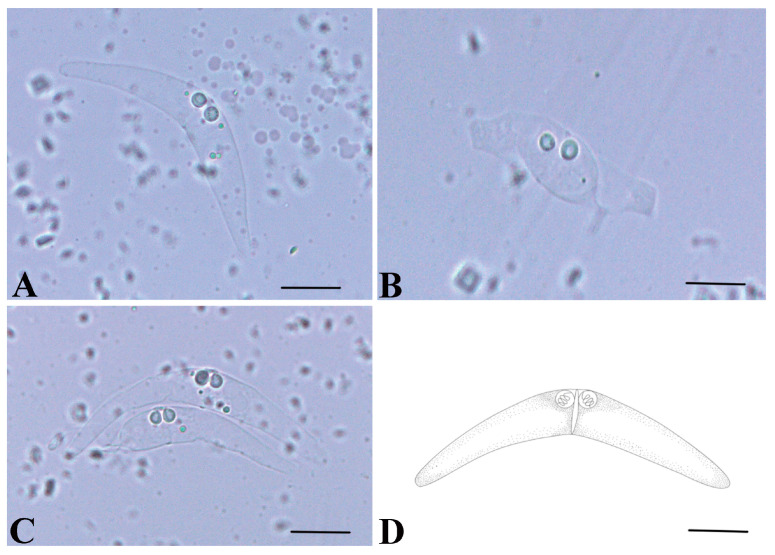
Photomicrographs of *Ceratomyxa nemiptera* sp. nov. from the gallbladder of *Nemipterus virgatus*. (**A**) mature spore; (**B**) immature spore; (**C**) disporic plasmodium of *Ceratomyxa nemiptera* sp. nov.; (**D**) Line drawings of mature spores of *Ceratomyxa nemiptera* sp. nov., Scale bar 10 μm.

Numerous mature and immature myxospores of *C. nemiptera* sp. nov. were observed in the bile within the gallbladder of *N. virgatus*. Immature myxospores exhibited irregular protoplasmic shapes, and the disporic plasmodium possessed four developing polar capsules (Figure 2B,C). Mature myxospores were crescent-shaped, measuring 6.2 ± 0.6 (5.4–6.9) μm in length and 44.8 ± 4.6 (38.5–53.1) μm in thickness (Figure 2A,D; Table 1). The myxospore valves were symmetrical, divided into two equal valves by a vertical suture, gradually tapering laterally with rounded ends, consistent with the typical characteristics of the genus *Ceratomyxa*. Each myxospore possessed two sub-spherical polar capsules, situated near the anterior end and closely appressed to the suture. The polar capsules measured 2.8 ± 0.2 (2.4–3.1) μm in length and 2.3 ± 0.2 (1.9–2.6) μm in width (Figure 2A,D; Table 1). The polar filaments were coiled in 2–3 turns. The posterior angle of the myxospore measured 131.6° ± 14.6° (103.3–153.0°) (Figure 2A,D; Table 1).

### 3.3. Remarks

Among all previously described species of *Ceratomyxa*, the morphology of *C. nemiptera* sp. nov. most closely resembles that of *Ceratomyxa fistulariae* Kpatcha, Diebakate, Faye & Toguebaye, 1996, *Ceratomyxa anko* Freeman, Yokoyama & Ogawa, 2008, *Ceratomyxa protopsettae* Fujita, 1923, *Ceratomyxa draconis* Azizi, Yemmen, Rangel, Santos & Bahri, 2020, *Ceratomyxa macapaensis* Bittencourt, 2022, *Ceratomyxa mandii* Araújo, 2022.

*Ceratomyxa fistulariae* can be distinguished from the new species by its markedly greater myxospore length (5.4–6.9 vs. 10–12 μm). In addition, the polar capsules of *C. fistulariae* are pear-shaped and considerably larger, whereas those of the new species are sub-spherical and smaller (2.4–3.1 vs. 4.5–5.5 μm) (Table 1). The host of *C. fistulariae* is *Fistularia petimba* Lacepède, 1803, collected from the Atlantic Ocean off Senegal, while the new species was found in *N. virgatus* from the East China Sea. *C. anko* possesses greater myxospore length and larger polar capsules than the new species (myxospore length: 5.4–6.9 vs. 9.7–11.9 μm; polar capsule length: 2.4–3.1 vs. 4.1–5.3 μm). Moreover, the end of valves of *C. anko* are more rounded compared with those of the new species (Table 1). Additionally, *C. anko* was described from the host *Lophius litulon* Jordan, 1902, collected off Fukushima, Japan, which differs from the host of the new species, *N. virgatus*, from the East China Sea. *C. protopsettae* differs from the new species in having larger myxospores and polar capsules (myxospore size: 5.4–6.9 × 38.5–53.1 μm vs. 10–12 × 50–65 μm; polar capsule size: 2.8 ± 0.2 (2.4–3.1) μm vs. 4.15 ± 0.34 μm). In addition, *C. protopsettae* possesses a greater number of polar filament turns (5–6 vs. 2–3) (Table 1). Additionally, *C. protopsettae* was described from *Paralichthys olivaceus* Temminck & Schlegel, 1846, collected off the East Sea of Korea, whereas the new species was found in *N. virgatus* from the East China Sea. Among the six most morphologically similar congeners, *C. draconis* is the only species showing the highest SSU rDNA sequence similarity (92.49%) to the new species (Table 1). *C. draconis* differs from the new species in having thinner myxospores (38.5–53.1 μm vs. 28.8–32.8 μm) and larger polar capsules (2.4–3.1 × 1.9–2.6 μm vs. 3.6–4.0 μm), although the range of its posterior angle partially overlaps with that of the new species (103.3–153.0° vs. 120–156°) (Table 1). *C. draconis* parasitizes *Trachinus draco* Linnaeus, 1758 from the Gulf of Bizerte, Tunisia, whereas the new species was found in *N. virgatus* from the East China Sea.

*Ceratomyxa macapaensis* and *C. mandii* differ markedly from the new species in their ecological and morphological characteristics. Both species are freshwater taxa, whereas the new species is marine, with distinct host and locality associations (Table 1). In addition, the myxospores and polar capsules of *C. macapaensis* and *C. mandii* are distinctly smaller than those of the new species, and *C. mandii* further differs in having a markedly larger posterior valve angle (Table 1).

Compared with the six morphologically most similar congeners described above, *Ceratomyxa* species recorded from China are generally less similar to the new species. Among the Chinese taxa, only *Ceratomyxa mai* Yang, Huang, Atkinson, Bartholomew, Ma & Zhao, 2023, *Ceratomyxa saurida* Zhao, Al-Farraj, Al-Rasheid & Song, 2015, and *Ceratomyxa siganicola* Zhang, Zhao, Yang & Yang, 2019 show a certain degree of morphological resemblance.

*Ceratomyxa mai* can be distinguished from the new species by its markedly greater myxospore length (8.1–9.9 vs. 5.4–6.9 μm) and considerably thinner myxospore (17.3–24.7 vs. 38.5–53.1 μm). In addition, the polar capsules of *C. mai* are wider than those of the new species (2.4–3.3 vs. 1.9–2.6 μm). Although the range of its posterior angle partially overlaps with that of the new species (125.8–158.2° vs. 103.3–153.0°). The host of *C. mai* is *Saurida elongata* Temminck & Schlegel, 1846, whereas the host of the new species is *N. virgatus*. *Ceratomyxa saurida* differs from the new species in having greater myxospore length (8.0–10.6 vs. 5.4–6.9 μm) and similar myxospore thickness (38.1–54.6 vs. 38.5–53.1 μm). In addition, the polar capsules of *C. saurida* are longer than those of the new species (3.0–3.7 vs. 2.4–3.1 μm). Although the range of its posterior angle partially overlaps with that of the new species (138.1–176.3° vs. 103.3–153.0°). The host of *C. saurida* is *Saurida elongata*, while the host of the new species is *N*. *virgatus*. *Ceratomyxa siganicola* can be distinguished from the new species by its markedly thinner myxospore (16.0–22.1 vs. 38.5–53.1 μm), although the range of myxospore length partially overlaps (4.8–6.5 vs. 5.4–6.9 μm). In addition, the polar capsules of *C. siganicola* are smaller than those of the new species (2.1–3.0 vs. 2.4–3.1 μm). Although the range of its posterior angle partially overlaps with that of the new species (175.2–178.4° vs. 103.3–153.0°). The host of *C. siganicola* is *Siganus fuscescens* Houttuyn, 1782, while the new species infects *N. virgatus*.

### 3.4. Molecular and Phylogenetic Analysis

A partial SSU rDNA sequence of *C. nemiptera* sp. nov., 1426 bp in length, was successfully amplified and sequenced from the type host and has been deposited in GenBank under accession number PX637768. Molecular analysis revealed that this sequence exhibited the highest similarity (93.56%) and the shortest genetic distance (0.0637) to *Ceratomyxa arcuata* Thélohan, 1892 (KJ419344), followed by *Ceratomyxa cretensis* Kalatzis, Kokkari & Katharios, 2013 (JX869942) with 93.38% similarity and 0.0681 genetic distance (Table 2). Among the species that share morphological resemblance with *C. nemiptera* sp. nov., *C. draconis*, *C. anko*, *C. mandii* and *C. macapaensis* possess publicly available SSU rDNA sequences. Comparative analysis revealed that the *C. nemiptera* sp. nov. showed the highest sequence similarity (92.49%) and the smallest genetic distance (0.0751) with *C. draconis*, while it exhibited the lowest sequence similarity (75.74%) and largest genetic distance (0.2790) with *C. mandii* (Table 2).

**Figure 3:**
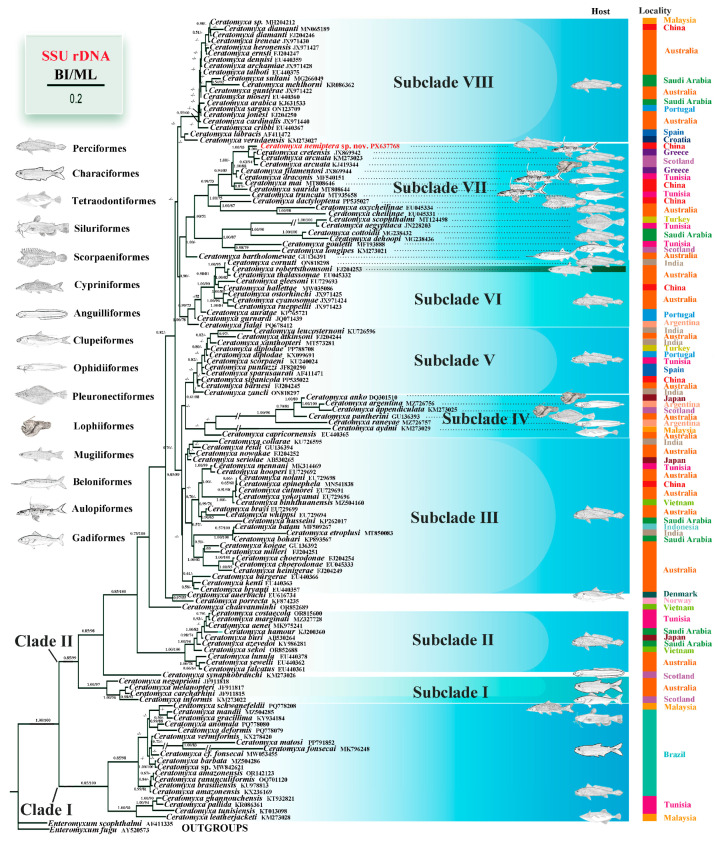
Phylogenetic tree generated by BI and ML based on the SSU rDNA gene sequences of *Ceratomyxa nemiptera* sp. nov., The node indicates the support value, with “-” indicating values less than 50%; the number after the species name indicates the GenBank accession number. “//” indicates that the branch is drawn two-third of the original length.

Phylogenetic trees based on the SSU rDNA sequences were reconstructed using both ML and BI approaches, and the resulting topologies were consistent. The phylogenetic tree was resolved into two major clades, Clade I and Clade II (Figure 3). The Clade I represents an early-diverging lineage comprising *Ceratomyxa* species parasitizing freshwater, brackish, and some marine fish hosts. Clade II was subdivided into eight subclades, encompassing species infecting marine and brackish-water fishes (Figure 3). *C. nemiptera* sp. nov. was positioned within a later-diverging lineage, Subclade VII, forming a sister-group relationship with a clade containing *C. arcuata* and *C. cretensis* (Figure 3).

## 4. Discussion

Recent studies have demonstrated a correlation between the phylogeny of myxosporeans and the taxonomic affiliation of their hosts, with closely related myxosporean species typically infecting hosts belonging to the same taxonomic groups [42,43,44,45,46]. The results of this study are consistent with this pattern. As shown in the phylogenetic tree, all parasites within Subclades II, III, V, and VIII each infect hosts that clearly belong to the order Perciformes (Figure 3). These derived lineages not only display a relatively narrow host range but also demonstrate a distinct evolutionary tendency toward host specificity. However, not all lineages follow this pattern. For instance, parasites within Subclades IV and VII are phylogenetically closely related (Figure 3), yet their hosts belong to different orders. In particular, within Subclade VII, the new species infects a perciform host (*N*. *virgatus*), whereas its close relatives parasitize hosts from the orders Lophiiformes, Aulopiformes, Ophidiiformes, and Clupeiformes.

Within Subclade VI, all species parasitize perciform hosts except *Ceratomyxa robertsthomsoni* Gunter, Whipps & Adlard, 2009 (FJ204253), which infects a mugiliform host (Figure 3). This observation may suggest the possibility of an adaptive “host-switching” strategy during the evolutionary diversification of *Ceratomyxa* species [14,47,48,49]. Such host-switching events may represent an adaptive mechanism that enables certain species to expand their host range by overcoming the taxonomic boundaries of their ancestral host groups. It is possible that *C*. *robertsthomsoni* has undergone a host shift from Perciformes to Mugiliformes, thereby broadening its potential host spectrum. This hypothesis suggests that host-switching may occur through different evolutionary pathways. Two scenarios could be envisaged: one in which the parasite retains the ancestral host while adapting to a new one, and another in which it abandons the original host and parasitizes only the new host. However, the apparent absence of the “original host” does not necessarily imply its true absence [50].

We also found that, in some clades, closely related *Ceratomyxa* species were distributed within the same geographical region. For instance, in Subclades III, VI, and VIII, most *Ceratomyxa* species originated from Australia and exhibited relatively close phylogenetic relationships within their respective subclades, suggesting that their evolutionary relationships may be partly influenced by geographic distribution. However, these subclades also include species from other regions that are phylogenetically close to the Australian taxa (Figure 3). Such a pattern corresponds to what has been observed in free-living organisms, whose genetic and evolutionary relationships often reflect geographic structuring, whereby taxa sharing a common geographic origin tend to be more closely related [51,52,53]. Yet, for parasitic organisms, geographical proximity is not necessarily the sole or dominant determinant of genetic relatedness. The phylogeny of parasites is frequently shaped by the dispersal capacity and lineage affinities of their hosts and can also be affected by host-switching events, complex life cycles involving multiple hosts or vectors, and human-mediated translocation across regions. These processes may weaken or obscure the otherwise direct correspondence between phylogenetic relatedness and geographic distance [54,55,56].

In Clade I (Figure 3), *Ceratomyxa* species parasitizing freshwater fishes (e.g., *C. mandii* (MZ504285), *Ceratomyxa fonsecai* Silva, 2020 (MK796248)); brackish-water fishes (e.g., *Ceratomyxa tunisiensis* Thabet, Mansour, Al Omar & Tlig-Zouari, 2015 (KT013098)) and marine fishes (e.g., *Ceratomyxa ghannouchensis* Thabet, Abdel-Baki, Harrath & Mansour, 2019 (KT932821), *Ceratomyxa pallida* Thélohan, 1895 (KR086361)) clustered together, forming the basal lineage of the *Ceratomyxa* clade. This group appears to represent an evolutionarily ancient lineage, consistent with previous findings [42,57]. These observations raise an intriguing question regarding the evolutionary origin of *Ceratomyxa*: did the genus originate in marine or freshwater environments? Earlier studies have suggested that myxosporeans originated in the marine environment [3,49]. A possible explanation is that the ancestral *Ceratomyxa* lineage arose in the ocean and subsequently colonized freshwater habitats through marine transgression events, during which multiple adaptive radiations occurred, leading to the present phylogenetic pattern [2].

The host species of *C. nemiptera* sp. nov., *N. virgatus*, is an economically important demersal fish widely distributed in the East China Sea and along the southeastern coast of China, where it contributes substantially to regional marine fisheries [17]. Although no obvious clinical signs or gross pathological alterations were observed in the infected hosts examined in the present study, previous studies have shown that myxosporean species infecting the gallbladder, including members of the genus *Ceratomyxa*, are generally characterized by low pathogenicity and often occur without apparent disease symptoms in their natural hosts [12,14,15].

Gallbladder infections by ceratomyxids are commonly reported in marine teleosts and are frequently considered to have limited direct pathological impact; however, such infections may still represent a chronic physiological burden to the host, particularly under environmental stress or high exploitation pressure [12,15]. Importantly, myxozoans, including species of *Ceratomyxa*, are currently not known to be zoonotic and do not pose a direct risk to human health [1,3]. Nevertheless, parasitic infections in commercially valuable fishes may indirectly influence seafood quality and market perception, as demonstrated for other myxozoans such as *Kudoa* spp., which are known to affect flesh quality and marketability [58]. Therefore, continued monitoring and accurate identification of myxosporean parasites in economically important marine fishes remain essential for effective fisheries management and the sustainability of marine resources.

## 5. Conclusions

In summary, *Ceratomyxa nemiptera* sp. nov. is described as a new myxosporean species infecting the gallbladder of *Nemipterus virgatus* from the East China Sea, based on an integrative analysis combining morphological characteristics, host and infection-site specificity, and SSU rDNA sequence data. Both morphometric comparisons and molecular evidence clearly distinguish this species from all previously described congeners. Phylogenetic analyses place *C. nemiptera* sp. nov. within a later-diverging lineage of the genus *Ceratomyxa*, forming a sister-group relationship with *C. arcuata* and *C. cretensis*, suggesting a close evolutionary affinity with marine congeners parasitizing teleost hosts. The discovery of this species expands current knowledge of *Ceratomyxa* diversity in the East China Sea and contributes to a better understanding of the evolutionary relationships and host associations within this complex genus. This study further highlights the importance of integrative morphological and molecular approaches for accurate species delimitation in myxosporean parasites.

## Figures and Tables

**Table 1:** Comparison of the morphometry or morphology of *Ceratomyxa nemiptera* sp. nov. with the similar species (all measurements are provided in μm).

Species	SL	ST	PCL	PCW	PFT	PA (°)	Host	Locality	References
*Ceratomyxa nemiptera* sp. nov.	6.2 ± 0.6 (5.4–6.9)	44.8 ± 4.6 (38.5–53.1)	2.8 ± 0.2 (2.4–3.1)	2.3 ± 0.2 (1.9–2.6)	2–3	131.6 ± 14.6 (103.3–153.0)	*Nemipterus virgatus* Houttuyn, 1782	East China Sea, PR China	Present work
*Ceratomyxa fistulariae* Kpatcha, Diebakate, Faye & Toguebaye, 1996	10.2 (10–12)	39.6 (38.8–40)	5.2 (4.5–5.5)	-	-	-	*Fistularia petimba* Lacepède, 1803	Atlantic Ocean, Senegal	[36]
*Ceratomyxa anko* Freeman, Yokoyama & Ogawa, 2008	10.8 (9.7–11.9)	41.9 (36.9–47.2)	4.6 (4.1–5.3)	-	-	-	*Lophius litulon* Jordan, 1902	Fukushima Prefecture, Japan	[37]
*Ceratomyxa draconis* Azizi, Yemmen, Rangel, Santos & Bahri, 2020	7.4 ± 0.77 (6.4–8.0)	30.8 ± 1.65 (28.8–32.8)	3.3 ± 0.2 (3.6–4.0)	-	-	120–156	*Trachinus draco* Linnaeus, 1758	Bay of Bizerte, Tunisia	[38]
*Ceratomyxa protopsettae* Fujita, 1923	11.64 ± 0.95 (10–12)	46.63 ± 5.8 (50–65)	4.15 ± 0.34	-	5–6	-	*Paralichthys olivaceus* Temminck & Schlegel, 1846	East Sea, South Korea	[39]
*Ceratomyxa macapaensis* Bittencourt, 2022	4.2 ± 0.5	22.75 ± 0.3	1.86 ± 0.3	1.63 ± 0.1	3–4	-	*Mesonauta festivus* Heckel, 1840	Piririm River, Amapá, Brazil	[40]
*Ceratomyxa mandii* Bruno, 2022	4.6 ± 0.5 (3.4–5.5)	31.2 ± 2.3 (26.2–36.3)	1.8 ± 0.3 (1.0–2.5)	1.9 ± 0.3 (1.2–2.4)	3–4	162 ± 10.4 (143–178)	*Pimelodina flavipinnis* Steindachner, 1876	Amazon River, Amazonas, Brazil	[41]
*Ceratomyxa mai* Yang, Huang, Atkinson, Bartholomew, Ma & Zhao, 2023	9.2 ± 0.5 (8.1–9.9)	20.9 ± 1.9 (17.3–24.7)	2.6 ± 0.2 (2.4–2.9)	2.7 ± 0.2 (2.4–3.3)	-	142.2 ± 8.2 (125.8–158.2)	*Saurida elongata* Temminck & Schlegel, 1846	East China Sea, PR China	[10]
*Ceratomyxa saurida* Zhao, Al-Farraj, AL-Rasheid & Song, 2015	9.0 ± 0.7 (8.0–10.6)	42.5 ± 4.0 (38.1–54.6)	3.3 ± 0.3 (3.0–3.7)	-	-	156.8 ± 11.4 (138.1–176.3)	*Saurida elongata* Temminck & Schlegel, 1846	East China Sea, PR China	[10]
*Ceratomyxa siganicola* Zhang, Zhao, Yang & Yang, 2019	5.6 ± 0.5 (4.8–6.5)	19.1 ± 1.8 (16.0–22.1)	2.7 ± 0.2 (2.1–3.0)	-	-	177.1 ± 0.5 (175.2–178.4)	*Siganas fuscescens* Houttuyn, 1782	East China Sea, PR China	[7]

Note: SL means spore length; ST means spore thickness; PCL means polar capsule length; PCW means polar capsule width; PFT means polar filament turns; PA means posterior angle; - means no description or no data; The symbol ° stands for degrees. All measurements are given in micrometers, data are presented as mean ± standard deviation (range), unless otherwise stated.

**Table 2:** An analysis of genetic distance and similarity between *Ceratomyxa nemiptera* sp. nov. and most morphologically similar congeners based on SSU rDNA sequences.

Species	1	2	3	4	5	6	7
*Ceratomyxa nemiptera* sp. nov.		93.56%	93.38%	92.49%	78.88%	75.74%	76.86%
*Ceratomyxa arcuata*	0.0637		97.55%	93.28%	79.90%	78.32%	77.72%
*Ceratomyxa cretensis*	0.0681	0.0151		92.46%	78.70%	76.52%	77.85%
*Ceratomyxa draconis*	0.0751	0.0643	0.0738		78.52%	74.93%	76.61%
*Ceratomyxa anko*	0.2320	0.2000	0.2190	0.2400		70.52%	72.83%
*Ceratomyxa mandii*	0.2790	0.2410	0.2670	0.2850	0.3420		93.45%
*Ceratomyxa macapaensis*	0.2300	0.2200	0.2250	0.2370	0.2980	0.6400	

## Data Availability

All the datasets generated or analyzed during this study are included in this article.
